# Supplementary material for: The Efficacy of Primavera, a Prevention Programme on Alcohol and Tobacco Use among 10–12-Year-Old Schoolchildren: A Randomized Controlled Cluster Study
Source: Int J Environ Res Public Health. 2021 Apr 7;18(8):3852. doi: 10.3390/ijerph18083852 (PMC8067627; doi:10.3390/ijerph18083852)
Supplement: Supplementary file 1 [file ijerph-18-03852-s001.pdf]

Table S1: Distribution of potential confounders between intervention and control groups at baseline

Values are numbers (percentages) unless stated otherwise	Primavera group	Control group	OR (95%CI)
All subjects	283(52)	265 (48)	
**Social-economic characteristics**			
Mean (SD, Median) age	10.60 (0.35) 10.59	10.56 (0.40) 10.50	NA
Boys	140 (52)	126 (52)	1.01 [0.71-1.43]
Individual bedroom	213 (77)	190 (73)	1.21 [0.82-1.79]
Two cars or more in the household	172 (61)	143 (55)	1.31 [0.93-1.85]
Trip abroad during the last holiday	152 (55)	158 (61)	0.77 [0.55-1.09]
**Hobbies**			
Reading out-of-school books	210 (75)	204 (79)	0.82 [0.55-1.23]
Outings to parks and museums	174 (63)	184 (72)	**0.66 [0.46-0.95]**
Playing upon an instrument of music	47 (17)	51 (20)	0.82 [0.53-1.26]
Doing out-of-school sports	201 (78)	170 (75)	1.22 [0.80-1.87]
Playing video games (on computer, console or phones)	268 (99)	239 (98)	1.40 [0.37-5.72]
**Time spent watching screens during (TV, videos, "You tube" or similar devises, DVD…)**			
No more than 2 hours spent watching screens during the week	195 (70)	200 (77)	0.70 [0.48-1.03]
No more than 2 hours spent watching screens during the week-end	123 (44)	151 (58)	**0.57 [0.41-0.80]**
**Self-perception on school results**			
"The teacher finds good or very good my school results compared to those of my schoolmates"	213 (77)	174 (67)	**1.64 [1.13-2.41]**
**Bullying in school**			
Never bullied in last 2 months or only happened once or twice	217 (80)	218 (83)	0.81 [0.52-1.26]
**Family habits**			
Having a parent using tobacco	173 (62)	139 (56)	1.30 [0.92-1.85]
Having a parent using alcohol	227 (82)	179 (71)	**1.82 [1.21-2.74]**
Talking about tobacco at home with his/her parents	55 (20)	59 (22)	0.86 [0.57-1.30]
Talking about alcohol at home with his/her parents	53 (19)	54 (21)	0.92 [0.60-1.41]
Talking about tobacco at home with a brother or a sister	15 (5)	22 (8)	0.63 [0.31-1.23]
Talking about alcohol at home with a brother or a sister	18 (7)	20 (8)	0.84 [0.43-1.62]
**Remembering having received a prevention intervention**			
Remembering having received information in school about alcohol	42 (15)	31 (12)	1.32 [0.80-2.18]
Remembering having received information in school about tobacco	41 (15)	28 (11)	1.45 [0.87-2.44]
Remembering having received information in school about another substance	35 (13)	30 (11)	1.12 [0.67-1.90]

Table S2: Distribution of potential confounders between intervention and control groups at 6 months from the start of the interventions (follow-up time point 1)

**Values are numbers (percentages) unless stated otherwise**	**Primavera group**	**Control group**	**OR (95%CI)**
All subjects	273(52)	255(48)	
**Social-economic characteristics**			
Mean (SD, Median) age	11.00 (0.40) 11.00	10.95 (0.36) 10.92	NA
Boys	126 (48)	122 (49)	0.96 [0.67-1.35]
Individual bedroom	216 (80)	186 (74)	1.37 [0.91-2.07]
Two cars or more in the household	186 (68)	158 (62)	1.29 [0.91-1.86]
Trip abroad during the last holiday	152 (57)	156 (61)	0.82 [0.58-1.17]
**Hobbies**			
Reading out-of-school books	192 (71)	197 (78)	0.71 [0.48-1.06]
Outings to parks and museums	184 (69)	201 (80)	**0.60 [0.38-0.85]**
Playing upon an instrument of music	40 (15)	51 (20)	0.69 0.44-1.09]
Doing out-of-school sports	187 (78)	177 (78)	1.00 [0.65-1.54]
Playing video games (on computer, console or phones)	255 (98)	234 (98)	0.91 [0.26-3.05]
**Time spent watching screens during (TV, videos, "You tube" or similar devises, DVD…)**			
No more than 2 hours spent watching screens during the week	173 (64)	188 (74)	**0.64 [0.44-0.92]**
No more than 2 hours spent watching screens during the week-end	176 (65)	181 (71)	0.76 [0.52-1.09]
**Self-perception on school results**			
"The teacher finds good or very good my school results compared to those of my schoolmates"	217 (81)	183 (72)	**1.63 [1.08-2.46]**
**Bullying in school**			
Never bullied in last 2 months or only happened once or twice	222 (83)	209 (82)	1.04 [0.66-1.63]
**Family habits**			
Having a parent using tobacco	160 (60)	141 (56)	1.18 [0.83-1.67]
Having a parent using alcohol	204 (76)	178(71)	1.33 [0.90-1.97]
Talking about tobacco at home with his/her parents	86 (32)	65 (26)	1.37 [0.94-2.02]
Talking about alcohol at home with his/her parents	72 (27)	47 (18)	**1.62 [1.07-2.46]**
Talking about tobacco at home with a brother or a sister	24 (9)	25 (10)	0.89 [0.49-1.61]
Talking about alcohol at home with a brother or a sister	24 (9)	21 (8)	1.09 [0.59-2.02]
**Remembering having received a prevention intervention**			
Remembering having received information in school about alcohol	140 (58)	97 (38)	**2.21 [1.54-3.17]**
Remembering having received information in school about tobacco	150 (62)	97 (39)	**2.60 [1.81-3.75]**
Remembering having received information in school about another substance	124 (51)	78 (32)	**2.22 [1.54-3.21]**

Table S3: Distribution of potential confounders between intervention and control groups at 18 months from the start of the interventions (follow-up time point 2)

**Values are numbers (percentages) unless stated otherwise**	**Primavera group**	**Control group**	**OR (95%CI)**
All subjects	197(55)	163(45)	
**Social-economic characteristics**			
Mean (SD, Median) age	12.04 (0.36) 12.08	11.97 (0.34) 12.00	NA
Boys	90 (47)	80 (51)	0.86 [0.56-1.31]
Individual bedroom	154 (78)	133 (82)	0.81 [0.48-1.36]
Two cars or more in the household	132 (67)	102 (63)	1.23 [0.80-1.91]
Trip abroad during the last holiday	124 (63)	103 (63)	1.00 [0.65-1.54]
**Hobbies**			
Reading out-of-school books	109 (57)	101 (62)	0.78 [0.51-1.20]
Outings to parks and museums	135 (70)	114 (70)	0.96 [0.61-1.52]
Playing upon an instrument of music	28 (14)	35 (22)	0.60 [0.35-1.04]
Doing out-of-school sports	122 (71)	104 (71)	0.99 [0.61-1.60]
Playing video games (on computer, console or phones)	183 (97)	149 (99)	0.41 [0.06-1.81]
**Time spent watching screens during (TV, videos, "You tube" or similar devises, DVD…)**			
No more than 2 hours spent watching screens during the week	123 (63)	96 (59)	1.17 [0.77-1.80]
No more than 2 hours spent watching screens during the week-end	105 (54)	87 (53)	1.02 [0.67-1.55]
**Self-perception on school results**			
"The teacher finds good or very good my school results compared to those of my schoolmates"	117 (59)	99 (61)	0.95 [0.62-1.44]
**Bullying in school**			
Never bullied in last 2 months or only happened once or twice	159 (84)	134 (86)	0.87 [0.47-1.57]
**Family habits**			
Having a parent using tobacco	113 (59)	77 (48)	**1.60 [1.05-2.44]**
Having a parent using alcohol	156 (82)	118 (73)	**1.67 [1.01-2.80]**
Talking about tobacco at home with his/her parents	56 (29)	43 (27)	1.12 [0.70-1.79]
Talking about alcohol at home with his/her parents	48 (25)	46 (29)	0.83 [0.52-1.33]
Talking about tobacco at home with a brother or a sister	17 (9)	17 (11)	0.81 [0.40-1.65]
Talking about alcohol at home with a brother or a sister	18 (9)	15 (9)	0.99 [0.48-2.06]
**Remembering having received a prevention intervention**			
Remembering having received information in school about alcohol	97 (54)	18 (11)	**9.33 [5.38-16.96]**
Remembering having received information in school about tobacco	90 (50)	15 (9)	**9.60 [5.37-18.21]**
Remembering having received information in school about another substance	63 (34)	16 (10)	**4.61 [2.59-8.64]**
